# Human Sprouty1 Suppresses Urokinase Receptor-Stimulated Cell Migration and Invasion

**DOI:** 10.1155/2013/598251

**Published:** 2013-09-12

**Authors:** Ahmed H. Mekkawy, David L. Morris

**Affiliations:** Department of Surgery, Cancer Research Laboratories, University of New South Wales, Sydney, NSW 2217, Australia

## Abstract

The urokinase-type plasminogen activator receptor (uPAR) has been implicated in several processes in tumor progression including cell migration and invasion in addition to initiation of signal transduction. Since uPAR lacks a transmembrane domain, it uses the interaction with other proteins to modulate intracellular signal transduction. We have previously identified hSpry1 as a partner protein of uPAR, suggesting a physiological role for hSpry1 in the regulation of uPAR signal transduction. In this study, hSpry1 was found to colocalize with uPAR upon stimulation with epidermal growth factor (EGF), urokinase (uPA), or its amino terminal fragment (uPA-ATF), implicating a physiological role of hSpry1 in regulation of uPAR signalling pathway. Moreover, hSpry1 was able to inhibit uPAR-stimulated cell migration in HEK293/uPAR, breast carcinoma, and colorectal carcinoma cells. In addition, hSpry1 was found to inhibit uPAR-stimulated cell invasion in breast carcinoma and osteosarcoma cell lines. Increasing our understanding of how hSpry1 negatively regulates uPAR-stimulated cellular functions may determine a distinctive role for hSpry1 in tumour suppression.

## 1. Introduction

The serine protease urokinase-type plasminogen activator (uPA) receptor (uPAR) has been implicated in extracellular matrix (ECM) proteolysis, initiation of signal transduction, and important cell functions including migration and invasion [1]. The active uPA consists of catalytic protease domain and the uPA amino terminal fragment (uPA-ATF) [2]. uPA-ATF contains the kringle domain and the growth factor-like domain (GFD) that contains the binding sequence for the receptor [2]. The uPAR protein has been shown to engage in multiple protein-protein interactions with other proteins such as vitronectin, integrins, and Mrj [3–5].

Sprouty (Spry) proteins have been identified as inhibitors of the receptor tyrosine kinase (RTK) including (epidermal growth factor receptor) EGFR [6]. The mammalian genome contains four SPRY genes (SPRY 1–4) encoding proteins with a conserved cysteine-rich region at the carboxyl terminus [7]. Due to its central role in Ras/MAP kinase pathway, SPRY may act as a putative tumor suppressor gene, and that loss of expression or function may allow the cell to be hypersensitive to growth signals [8]. Interestingly, the tumor suppressor gene, WT1, has been found to bind with SPRY1 promoter and regulate it during kidney development [9]. Prostate ductal hyperplasia and low-grade prostatic intraepithelial neoplasia (PIN) have been observed in adult mouse with concomitant Spry1 and Spry2 loss of function [10]. Additionally, Spry1 expression has been shown to be modulated in a variety of cancers. hSpry1 expression was downregulated in human prostate carcinoma [11, 12] and was decreased or absent in 15% of primary human prostate cancer and 42% of metastatic human prostate cancer [13]. Moreover, silencing of SPRY1 caused complete regression of established rhabdomyosarcoma xenograft in mice [14]. In addition, PCR studies showed downregulation of hSpry1 expression in more than 94% of breast cancer patient samples, with respect to normal tissues [8]. We have used previously yeast two-hybrid screening of breast cancer cDNA library to identify hSpry1 as a candidate protein that interacts with uPAR [15]. This protein-protein interaction may influence a range of biological functions. Here, we show for the first time that hSpry1 may negatively regulate uPAR-stimulated cell migration and invasion* in vitro*.

## 2. Materials and Methods

### 2.1. Materials and Plasmid Transfection

The goat anti-uPAR antibody was purchased from R&D Systems (number AF807, Minneapolis, MN, USA). The mouse anti-human Spry1 antibody (A01) was purchased from Abnova Corporation, Taiwan. The hSpry1 DNA construct pCDNA3.1/hSpry1 was a gift from Dr. Bernard Kwabi-Addo (Department of Pathology, Baylor College of Medicine, Baylor Plaza, Houston, TX, USA). The recombinant analog EGF was obtained from Sigma Aldrich (#E-4269, St. Louis, MO, USA). The recombinant human uPA was purchased from R&D systems (#1310-SE, Minneapolis, MN, USA). The recombinant human uPA-ATF was obtained from American Diagnostics (#146, Stamford, CT, USA). Cells were transfected with recombinant or control vector in addition to GFP plasmid to control transfection efficiency. Transfections were performed using GeneJuice (#70967, Novagen, EMD Bioscience, Merck KGaA, Darmstadt, Germany).

### 2.2. Cell Culture

The human embryonic kidney HEK293 cells stably transfected with uPAR were kindly provided by Dr. Ying Wei (University of California, San Francisco, CA, USA). The human breast cancer MDA-MB-231, the human colorectal carcinoma HCT116, and the human osteosarcoma Saos-2 cell lines were obtained from the American Type Culture Collection (ATCC). Cells were maintained in Dulbecco's modified Eagle's medium supplemented with 10% FCS and 1% antibiotics.

### 2.3. Indirect Immunofluorescence

Cells transfected with either pCDNA3.1/hSpry1 or pCDNA3.1 empty plasmid were seeded into sterilized glass cover slips. Cells were washed with PBS, fixed, and permeabilized with ice cold methanol for 10 min at −20°C. Cells were then washed, blocked with 1% BSA, and incubated with primary antibodies in 1% BSA, followed by Rhodamine-conjugated and FITC-conjugated secondary antibodies in 1% BSA. Nuclei were counterstained with propidium iodide (PI; Sigma). Cells were then washed, mounted, and visualized using confocal laser scanning microscopy (Olympus IX71 Laser Scanning Microscope) and 60x oil immersion lens.

### 2.4. Migration Assay

Cell migration was measured by using wound healing assay as previously described [16]. Briefly, cells were seeded into culture dishes and incubated for 24 h at 37°C. Cells were then transfected with either pCDNA3.1/hSpry1 or pCDNA3.1 empty plasmid and incubated to create confluent monolayer. A wound was created manually by scrapping the cell monolayer with a yellow pipette tip. After washing, the media were replaced with fresh ones. The first images were taken at four different focal areas in each well, and cells were stimulated with EGF, uPA, or uPA-ATF 100 ng/mL or left without stimulation as a control. Cells were incubated in the incubator, and the second images were taken at 18 h. These images were analyzed quantitatively by measuring the distance of cell migration in the wounded region.

### 2.5. Invasion Assay

Cell invasion assay is similar to cell migration assay; however, it requires cells first to enzymatically penetrate a barrier of an ECM or basement membrane extract and then to migrate through it. Here, to assess the effect of hSpry1 in regulating cell invasion, we used 24-well Transwell system with polycarbonate membranes of 8.0 *μ*m pore size (#3422, CORNING). Briefly, membranes were coated with 20 *μ*g/mL collagen IV at 4°C overnight. Transfected cells (2 × 10^5^/well) were seeded onto the upper-side chambers in 0.2 mL of serum-free DMEM medium, and 0.6 mL of the same medium containing 1% FCS was added onto the lower chamber. The cells were allowed to adhere for 1 h. Then, chemotaxis was induced by the addition of EGF, uPA, or uPA-ATF (100 ng/mL) to the lower chamber. Media containing 10% FCS and 1% FCS were used as positive and negative controls, respectively. At the end of the incubation period (18 h), cells remaining in the upper chamber were scraped. Cells that invaded through the membrane to the lower surface were Giemsa stained and counted in five different fields under the light microscope. 

### 2.6. Statistical Analysis

Data is expressed as the mean ± S.D, and, where appropriate, the Student's *t-*test was performed using GraphPad Prism V5.0 software (GraphPad Software Inc., La Jolla, CA, USA). Results were considered statistically significant when *P* < 0.05.

## 3. Results

### 3.1. hSpry1 Colocalizes with uPAR upon Stimulation with EGF, uPA, and uPA-ATF

**Figure 1:**
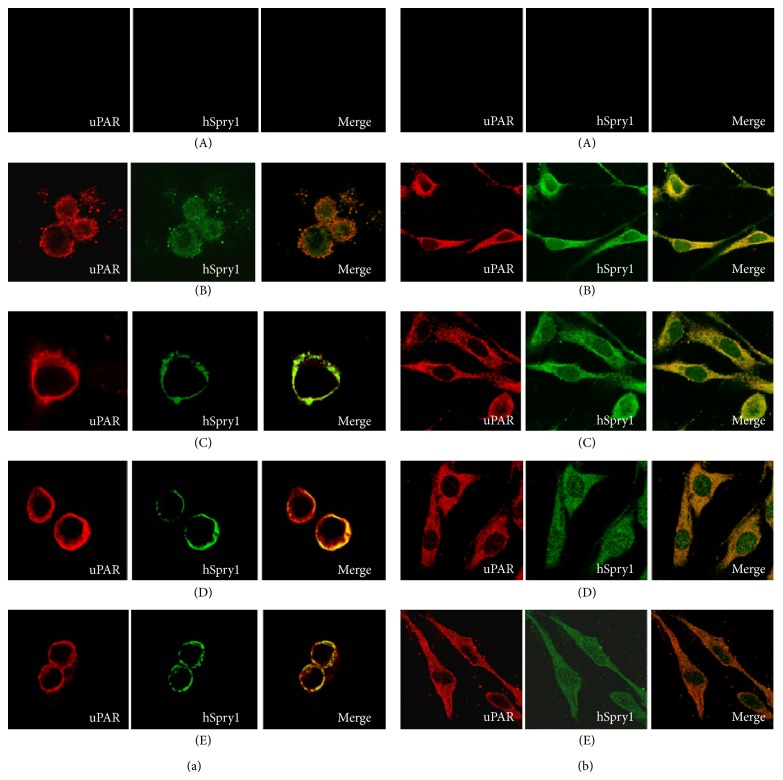
hSpry1 colocalizes with uPAR in cells with stimulated uPA and uPA-ATF. (a) HEK293/uPAR and (b) MDA-MB-231 cells overexpressing hSpry1 were kept as negative control (A) or serum-starved overnight (B) and then treated with 100 ng/mL of either EGF (C), uPA (D), or uPA-ATF (E) for 20 min. Cells were fixed and then immunostained with antibodies against uPAR (red) and hSpry1 (green), and colocalization appeared as a yellow colour. These cells were analysed using confocal laser scanning microscope and 60x oil immersion lens (final magnification 600x). Data are representative of 3 independent experiments.

The study of protein subcellular localization is important to elucidate protein function. We have previously shown the colocalization and physical interaction between uPAR and hSpry1 in cells cultured under growth media conditions [15]. Here, we used the immunofluorescence staining to investigate the expression and subcellular distribution of uPAR and hSpry1 upon stimulation of cells with EGF, uPA, or its amino terminal fragment. HEK293/uPAR and MDA-MB-231 cells both transfected with hSPry1 were employed for this experiment (Figure 1). Although, in MDA-MB-231 cells, uPAR seems to be colocalized with hSpry1 in the absence of any stimulation, greater colocalization between hSpry1 and uPAR has been observed upon stimulation with either EGF, uPA, or uPA-ATF in both HEK293/uPAR and MDA-MB-231 cells. These results suggest a physiological role for hSpry1 in the regulation of uPAR signal transduction. Hence, to investigate the role of hSpry1 as inhibitor of cell migration and invasion upon stimulation with EGF, uPA, and uPA-ATF was decided.

### 3.2. Overexpression of hSpry1 Inhibits Cell Migration upon EGF, uPA, and uPA-ATF Stimulation

**Figure 2:**
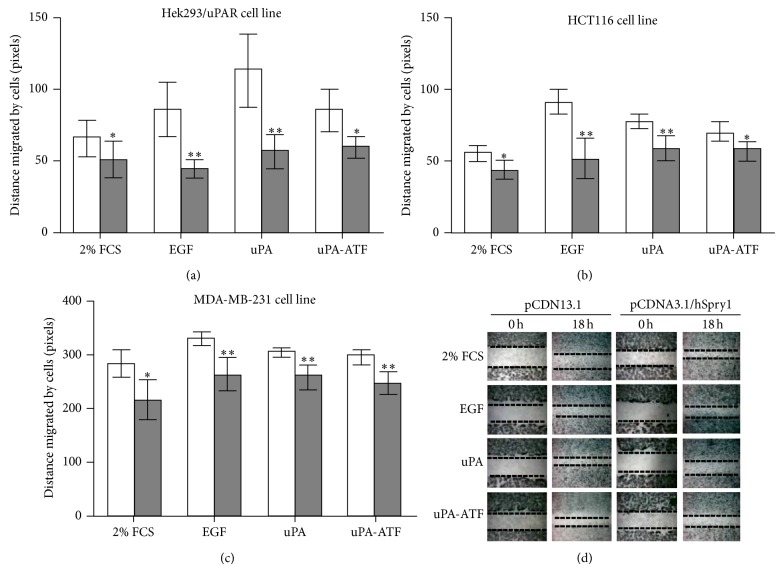
hSpry1 overexpression inhibits uPAR-stimulated cell migration. (a) HEK293/uPAR, (b) HCT-116, and (c) MDA-MB-231 cells were transfected with pCDNA3.1 (light bars) or pCDNA3.1/hSpry1 (dark bars) and examined using wound healing assay. (d) A monolayer of confluent cells (MDA-MB-231 cells in these photos) was wounded with a 200 *μ*L pipette tip, and the closure of the “scratch” was observed in the presence of either 10% FCS, 1% FCS, or 100 ng/mL of EGF, uPA, or ATF. Photographs taken immediately after the creation of a “scratch” are marked as 0 h. Photographs taken 18 h later demonstrate the differential extent of migration for the various cell lines. Cells migration was assessed by measuring the distance of the wounded region lacking cells in pixels cells after 18 h. Bars are mean ± SEM (*n* = 5), and significance was measured using unpaired *t*-test (^*^ *P* < 0.05; ^**^ *P* < 0.01). Data are representative of 3 independent experiments.

Previous reports show that uPAR promotes cell migration [17, 18]. Here, we used the wound healing assay to demonstrate the physiological impact of hSpry1 overexpression on uPAR-stimulated HEK293/uPAR, MDA-MB-231, and HCT-116 cell migration. As shown in Figure 2, the migration capacity of cells transfected with pCDNA3.1/hSpry1 compared to cells transfected with vector plasmid was significantly reduced (*P* < 0.05) in control group (2% FCS treated cells). Additionally, migration capacity of cells transfected with pCDNA3.1/hSpry1 and treated with EGF and uPA was significantly inhibited (*P* < 0.01) when compared to pCDNA3.1 transfected cells. Cells treated with uPA-ATF showed a significant reduction (*P* < 0.05) in the migration capacity of HEK293/uPAR and HCT-116 cells transfected with hSpry1 versus control group. However, MDA-MB-231 cells transfected with hSpry1 showed a significant inhibition (*P* < 0.01) in the migration capacity.

### 3.3. Overexpression of hSpry1 Inhibits Cell Invasion upon EGF, uPA, and uPA-ATF Stimulation

**Figure 3:**
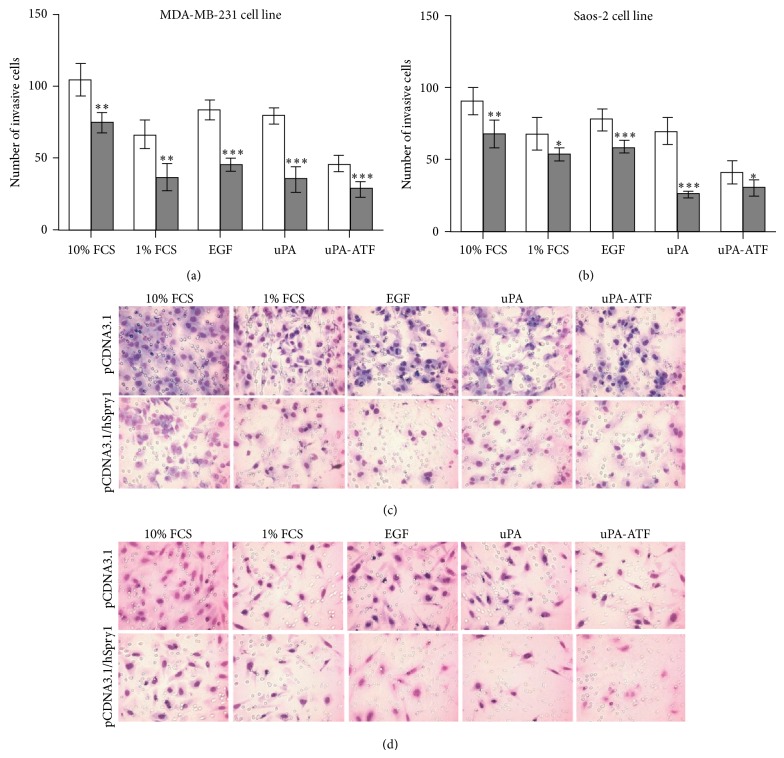
hSpry1 overexpression inhibits uPAR-stimulated tumor cell invasion. (a) MDA-MB-231 and (b) Saos-2 cells were transfected with pCDNA3.1 (light bars) or pCDNA3.1/hSpry1 (dark bars), placed in Transwell inserts, and were exposed to either 10% FCS, 1% FCS, or 100 ng/mL of EGF, uPA, or ATF. Cell invasion was assessed by counting (c) MDA-MB-231 and (d) Saos-2 cells on the membranes after 18 h. Bars are mean ± SEM (*n* = 5), and significance was measured using unpaired *t*-test (^*^ *P* < 0.05; ^**^ *P* < 0.01; ^***^ *P* < 0.001). Data are representative of 3 independent experiments.

We next used *in vitro* cell invasion assay to examine the ability of hSpry1 overexpression to inhibit uPAR-stimulated invasion. We chose as a model the invasive breast cancer MDA-MB-231 and osteosarcoma Saos-2 cell lines. In cells transfected with pCDNA3.1, EGF and uPA both increased MDA-MB-231 and Saos-2 cellular invasiveness (Figure 3). However, the catalytically inactive uPA-ATF caused a great suppression of MDA-MB-231 and Saos-2 cell invasiveness (Figure 3).

Compared to vector-transfected cells, MDA-MB-231 and Saos-2 cells transfected with pCDNA3.1/hSpry1 had significantly suppressed invasion capacity. This is reflected in the significant reduced number of invasive cells (*P* < 0.01) expressing hSpry1 upon stimulation with EGF and uPA (Figure 3). The comparison also revealed that overexpression of hSpry1 in the presence of ATF caused significant inhibition of MDA-MB-231 (*P* < 0.001) and Saos-2 (*P* < 0.05) cell invasiveness (Figure 3). 

## 4. Discussion

Overexpression of uPA-system components has been associated with aggressiveness in several types of cancer that offer attractive targets for development of new diagnostics and therapeutics. Many of the biological functions of uPAR necessitate interactions with other proteins on the cell surface, in particular, transmembrane proteins. The high lateral mobility of uPAR on the cell membrane may provide the mechanism by which it associates with other transmembrane receptors [19]. Additionally, the recent crystal structure model of the soluble form of uPAR revealed that uPA bounds to uPAR via a central cavity. This model left the external receptor surface free to bind and interact with other proteins [20]. In addition to the primary ligand uPA, a number of uPAR specific interactions have also been identified and are consistent with the varied functions regulated by uPAR including cell adhesion, cell migration, invasion, angiogenesis, and cancer metastasis. In fact, there is now evidence that uPAR can bind with the cell surface integrins [21–23], chemotactic receptors [24], EGFR [25], and Mrj [4]. Recently, we also reported hSpry1 as a candidate protein that interacts with uPAR [26], suggesting a physiological role for hSpry1 in the regulation of uPAR functions.

Proteins must be localized at their proper subcellular compartment to perform their desired role. Thus, knowing the subcellular localization of a protein can provide useful insights about its function. The subcellular localization of uPAR and hSpry1 proteins has been characterized. However, the colocalization of both proteins under stimulated conditions has not been identified. Hence, in this study, we investigated the subcellular localization and distribution of both proteins by indirect immunofluorescence. The mammalian Spry1 protein has been found predominantly in the perinuclear regions and in cytoplasmic vesicular structures of unstimulated cells [27]. However, upon stimulation with growth factors, the protein relocates to membrane ruffles, where it shows a partial overlap with the localization of caveolin-1 [12]. The significance of the membrane translocation of the Spry proteins might control its inhibitory activity and reflect the fact that several Spry-binding partners (Grb2 and Raf1) are located at membrane ruffles [26]. In addition, it has been found that both the tyrosine phosphorylation of Spry1 and the inhibition of RTK signaling by Spry1 occur at the plasma membrane, suggesting that the association with caveolin-1 might enhance Spry1 function [28]. Interestingly, uPAR has been also found to colocalize with caveolin [29, 30]. Moreover, it has been suggested that caveolin and uPAR may operate within adhesion sites to organize kinase rich lipid domains in proximity to integrins, promoting efficient signal transduction [31]. In MDA-MB-231 cells, uPAR seems to be colocalized with hSpry1 even in the absence of any stimulation. This suggests that the interaction between uPAR and Spry1 seems not to be correlated with the stimulation of uPAR in the cells. In addition, these findings implicate a physiological role for hSpry1 in uPAR regulation. This study was expanded by investigating the role of hSpry as an inhibitor of signal transduction initiated by EGF, uPA, or uPA-ATF stimulation.

Cellular migration is a key step in cancer invasion and metastasis. The uPA system plays a key role in these processes. Binding of uPA or uPA-ATF to uPAR at the cell surface stimulates cell migration [18]. In addition, *β*1-integrins interaction with uPAR was found to affect the intracellular pathways that regulate cell invasion [32]. EGFR can link the signal transduction initiated by uPAR to ERK pathway [33, 34], and EGFR inhibitors are capable of inhibiting the signal transduction generated by uPA [33]. In addition, EGFR activation is required for the invasion mediated by uPAR system [35, 36]. Furthermore, EGF stimulates uPAR expression and cell invasiveness in a variety of cancer cell lines [37, 38]. Moreover, uPA secretion and p56^LCK^-induced cell motility are mediated by activation of EGFR/ERK pathways [39]. Hence, EGFR appears to be a key molecule for uPAR-mediated tumour progression. Here, the results revealed that hSpry1 overexpression inhibits EGF-, uPA-, or uPA-ATF-stimulated cellular migration. In addition, hSpry1 overexpression suppressed the invasive capability of cancer cells to penetrate collagen layers in response to EGF, uPA, or uPA-ATF. These data indicate that the hSpry1 inhibits cell migration and invasion upon stimulation with EGF, uPA, or uPA-ATF. In conclusion, our results reveal an important role of hSpry1 expression in suppression of uPA system-stimulated migration and invasion in cancer cells. Further, these findings may explain why hSpry1 down-regulation or loss of function participates in cancer progression. The other uPAR candidate we have investigated previously was HAX1 [16]. Additional experiments to investigate the ability of hSpry1 and HAX1 to modulate uPAR functions like metastasis and angiogenesis may be performed in the future. To assess this, *in vitro *and *in vivo* models could be utilised. It is yet to be investigated whether or not all three proteins interact to form a large complex or compete with one another to affect uPAR functions. Increasing our understanding of the specific role of uPAR, hSpry1, and HAX1 in the molecular mechanism of cancer can assist clinically in the development of new tumour markers, which may permit more accurate determination of diagnosis in patients with cancer.
